# Rare germline variants in DNA repair-related genes are accountable for papillary thyroid cancer susceptibility

**DOI:** 10.1007/s12020-021-02705-1

**Published:** 2021-04-05

**Authors:** Catia Mio, Antonella Verrienti, Valeria Pecce, Marialuisa Sponziello, Giuseppe Damante

**Affiliations:** 1 Department of Medicine, University of Udine, 33100 Udine, Italy; 2 Department of Translational and Precision Medicine, “Sapienza” University of Rome, 00161 Rome, Italy

**Keywords:** Papillary thyroid cancer, DNA repair, Next-generation sequencing, Germline variants

## Abstract

**Background:**

Understanding the molecular mechanisms underlying papillary thyroid cancer (PTC) proved to be vital not only for diagnostic purposes but also for tailored treatments. Despite the strong evidence of heritability, only a small subset of alterations has been implicated in PTC pathogenesis. To this reason, we used targeted next-generation sequencing (NGS) to identify candidate variants implicated in PTC pathogenesis, progression, and invasiveness.

**Methods:**

A total of 42 primary PTC tissues were investigated using a targeted next-generation sequencing (NGS) panel enlisting 47 genes involved in DNA repair and tumor progression.

**Results:**

We identified 57 point mutations in 78.5% of samples (*n* = 32). Thirty-two somatic mutations were identified exclusively in known thyroid cancer genes (*BRAF, KRAS*, *NRAS*, and *TERT*). Unpredictably, 45% of the all identified mutations (*n* = 25) resulted to be germline, most affecting DNA repair genes. Interestingly, none of the latter variants was in the main population databases. Following ACMG classification, 20% of pathogenic/likely pathogenic and 68% of variant of unknown significance were identified.

**Conclusions:**

Overall, our results support the hypothesis that rare germline variants in DNA repair genes are accountable for PTC susceptibility. More data, including the segregation analysis in affected families, should be collected before definitely annotate these alterations and to establish their potential prognostic and treatment implications.

## Introduction

Understanding the molecular mechanisms underlying tumorigenesis is vital for the accurate diagnosis and use of personalized treatments. Previously, monogenic assays were commonly used to find molecular alterations in tumor mass populations [1]. Nowadays, the use of next-generation sequencing (NGS) technology ensures the simultaneous analysis of hundreds of genes of interest, using targeted sequencing panels.

Thyroid cancers (TC) are the most widespread malignancies of the endocrine system and represent ~1–1.5% of all tumor-related diseases [2]. TC typically progresses from thyroid nodules, which are prevalent in the general population and mainly related to old age. Although surgery solve most cases, in some cases the tumors exhibit an aggressive behavior. TC can be categorized by histology. Medullary TC accounts for about 5% of all cases and it rises from parafollicular C cells [3]. The remaining 95% of all TC cases are follicular cell-derived TC and they are classified in papillary (PTC), follicular, Hürthle, poorly differentiated, and anaplastic TC [4]. PTC alone accounts for 80% of all TCs.

Over the past decades, the application of molecular technologies has shed considerably light on the genetic abnormalities associated with TC, elucidating novel approaches to tumor diagnosis, risk assessment of tumor progression, and potential novel therapeutic strategies [5]. Well-known somatic alterations include single-nucleotide variants (SNVs) affecting proto-oncogenes (*BRAF, NRAS, HRAS, KRAS, EIF1AX*) and chromosomal rearrangements (*RET/PTC1, RET/PTC3, PAX8/PPARG*), which vary with the histologic subtype [6]. Notwithstanding the tremendous effort to better characterize PTC etiology, the molecular bases of tumor development are still elusive.

DNA repair is a gatekeeper set of pathways meant to preserve genomic integrity upon exposure to genotoxic agents. Impairments in these fine-tuned systems are correlated to cancer susceptibility. DNA repair proteins functionally interact with each other, within the same DNA repair pathway and across different pathways, establishing ground for additive or even multiplicative effects on DNA repair activity and, hence, cancer risk [7]. Moreover, the identification of novel prognostic markers is critical for the improvement of the risk stratification for cancer death and recurrence. We created a targeted NGS panel enlisting genes directly involved in DNA repair and some of the most important genes involved in tumor progression. The final aim was to identify SNVs that could play a role in cancer susceptibility and to discover new biomarkers of PTC progression and invasiveness to be used in the future, allowing the development of tailored PTC prevention policies, treatment possibilities and perhaps implementation of guidelines.

## Materials and methods

### Samples collection and DNA extraction

Informed consent was obtained from all individuals included in the study. Papillary thyroid tumors were obtained upon surgical resection at the Sapienza University of Rome Hospital. Immediately after surgery, tumor tissues were snap-frozen and stored in liquid nitrogen. Fresh-frozen tissue samples were reviewed by two different pathologists, who confirmed the diagnosis of PTC and excluded specimens in which tumor cells accounted for <60% of the total. Clinical data were collected by retrospective review of hospital charts, and tumors were staged according to the criteria of the AJCC/UICC TNM classification, 8th edition [8]. Tumors were risk-stratified based on clinical and histological data in accordance with the 2015 American Thyroid Association (ATA) risk of recurrence staging system [9]. Genomic DNA was isolated from surgical samples and peripheral blood as previously described [10].

### Library preparation and next-generation sequencing (NGS)

Genomic DNA from 42 tumor tissues was quantified using the Qubit dsDNA HS Assay Kit (Life Technologies). Barcoded libraries were generated from 0.5 to 10 ng of DNA per sample using the Ion AmpliSeq HiFi mix (Ion AmpliSeq Library Kit Plus, Thermo Fisher Scientific) and two premixed pools of 952 primer pairs (Thermo Fisher Scientific), according to manufacturer’s instructions. Gene panel list is shown in Supplementary Table 1. Clonal amplification of libraries was performed by emulsion PCR on an Ion Chef Instrument, as previously described [11]. Sequencing was performed with the Ion S5 GeneStudio Sequencer using the Ion 540 Chip kit and the Ion 540™ Kit-Chef (all Thermo Fisher Scientific).

### Data analysis and variant prioritization

The Variant Caller v5.12 was used to process data (Thermo Fisher Scientific). Annotation was performed with both Ion Reporter 5.12 (Thermo Fisher Scientific) and wANNOVAR, as previously described [12]. Briefly, somatic variants were called when a position was covered at least 500 times. High-quality variants were those with a depth of coverage (FDP) of ≥500, genotype quality scores of ≥30, a minimum alternate allele frequency of 5% (AF ≥ 5%), and absence of homopolymer regions (HRUN < 6). Finally, variants were prioritized based on their genomic location, with exclusion of intronic, intergenic, ncRNA-intronic, and UTR variants. Variant prioritization was based on population frequency, quality values, and functional consequences. Synonymous variants not affecting splice regions were excluded a priori. Variants were filtered based on their frequency among the European-descendent population (1000 Human Genomes Project, ESP6500SI, gnomAD, ExAC) and on clinical associations (NCBI dbSNP, ClinVar). In silico functional consequences were evaluated in seven databases (SIFT, Polyphen2_HVAR, Polyphen2_HDIV, LRT, MutationTaster, MutationAssessor, FATHMM, PROVEAN). After filtering and prioritization, missense, splice-site, stop-gain, stop-loss, and frameshift variants were retained for further evaluation. ACMG classification was used to annotate variants by the Varsome (https://varsome.com/) and wIntervar (http://wintervar.wglab.org/) online tools. The Catalogue Of Somatic Mutations In Cancer database was used to assess pathogenicity of somatic mutations (https://cancer.sanger.ac.uk/cosmic).

### Sanger sequencing

Candidate variants and *TERT* promoter mutations were assessed by Sanger sequencing as previously described [13]. PCR conditions and sequencing primers are available upon request. *TERT* mutational analysis was performed in samples with remaining DNA after NGS analysis.

### Statistical analysis

Data are reported as medians and range of values. Between-groups differences in categorical variables were analyzed using the Chi-square test or the Fisher exact test. Between-groups differences in continuous variables were assessed with the Mann–Whitney *U* test. *p* values lower than 0.05 were considered statistically significant. Statistical analyses were performed using GraphPad Prism version 8.0 software (GraphPad Software Inc., San Diego, CA, USA).

## Results

**Fig. 1:**
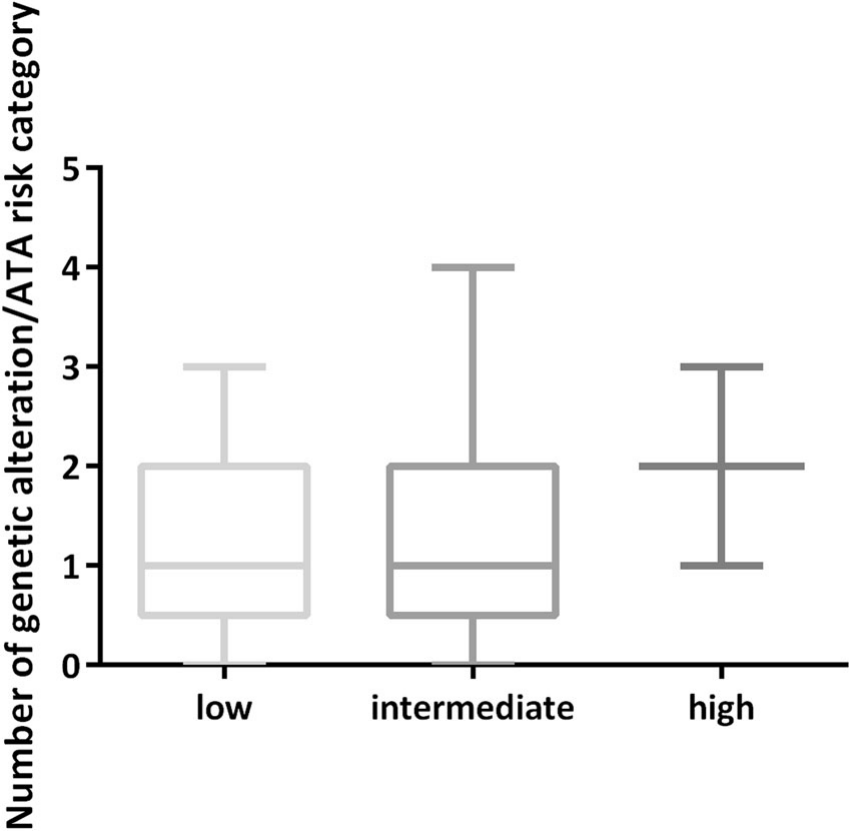
Association between the number of genetic alteration and clinical ATA category. No significant difference was assessed between the three ATA risk score-based categories

The aim of this study was to investigate the presence of novel point mutations and small insertions or deletions (indels) in genes related to DNA repair and to tumor progression in PTC, using a NGS approach. A total of 42 primary PTC tissues from 42 patients were investigated. Table 1 summarizes the characteristics of the patients enrolled in our cohort. Most of the cases were classic PTC (PTC-CT) (*N* = 31; 74%). The second largest histology subgroup was follicular variant PTC (PTC-FV) (*N* = 7; 17%), followed by: mixed PTC (*N* = 2; 5%), oxyphilic variant (*N* = 1; 2%), and trabecular variant (*N* = 1; 2%). The female:male ratio was 1.5:1 and 7% of cases were familial, defined as cases with two first- or second-degree relatives also affected with PTC. The 42 tissues were analyzed for mutations using small amount (0.5–10 ng) of DNA. Sequence coverage was assessed from the number and distribution of reads across the target DNA regions. Approximately 4.16 million mapped reads with the mean read length of 200 bp were generated. The mean depth of coverage was 4100X, with 96.7% reads on target. Overall, mutations were identified in 32 PTC specimens (78.5%). Fifty-seven single-nucleotide variants were detected in 18 coding genes. After filtering benign and likely benign SNVs, the median number of genetic alterations per ATA risk category was 2 (ranged from 0 to 4), which is consistent with most endocrine-related tumors having low mutational burden (Fig. 1). Overall, the totality of somatic mutations (*N* = 32) found in PTC specimens have been detected in *BRAF*, *KRAS*, and *NRAS* and possessed VAFs that ranged from 12 to 46%. The BRAF p.V600E mutation was confirmed as the most common hot-spot mutation in PTC (57%). Well-established *RAS* gene mutations (KRAS p.G12V, NRAS p.Q61K, and p.Q61R) were found in 5 PTCs (12%). *TERT* promoter mutations (*TERT* c. −124C > T (C228T) and c.-146C > T (C250T)) were identified in 12% of analyzed PTCs. In two patients *TERT* mutation was found in co-presence with BRAF p.V600E.

**Table 1:** Patients characteristics

Samples (Patients)	42 (42)
Sex^a^	
Female	23 (55%)
Male	16 (38%)
NA	3 (7%)
Average age at diagnosis (m ± SD)^a^	47 ± 15.9
Minimum	22
Maximum	84
Histology	
PTC-CT	31 (74%)
PTC-FV	7 (17%)
PTC-MIXED	2 (5%)
PTC-oxyphilic	1 (2%)
PTC-TRABECULAR	1 (2%)
Tumor size (mm), (m ± SD)^b^	13 ± 12.1
Minimum	3
Maximum	50
T stage	
T1a	14 (33.3%)
T1b	16 (38%)
T2	6 (14.3%)
T3	5 (12%)
T4	1 (2.4%)
N stage	
N0	19 (45%)
N1a	5 (12%)
N1b	10 (24%)
Nx	8 (19%)
M stage	
M0	23 (55%)
M1	1 (2%)
Mx	18 (43%)
ATA Risk of recurrence at diagnosis	
Low	17 (40.5%)
Intermediate	23 (55%)
High	2 (4.5%)
Evidence of disease at the last follow-up^c^	
Not evidence of disease	20
Biochemical evidence of disease	4
Structural evidence of disease	2
Positive family history^d^	
Yes	2 (7%)
No	25 (93%)

*SD* standard deviation, *NA* not available

^a^Three NA

^b^One NA

^c^16 NA

^d^Positive family history defined as cases with two first- or second-degree relatives also affected with PTC, 15 NA

The remaining alterations identified, included SNVs with an allelic frequency ranging between 42 and 58%, suggesting the possibility that these variants might be germline. To this reason, Sanger sequencing was performed on DNA extracted from patient’s blood samples, confirming the germline origin of these variants. All the aforementioned variants and the clinical features of the patients are reported in Table 2.

**Table 2:** Clinical features of patients harboring germline variants in DNA repair genes

Gene ID (Transcript ID)	Nucleotide change	Amino acid change	Clinical interpretation^a^	Other germline variants	Somatic mutations	Age at diagnosis	PTC variant	ATA risk	Outcome	Other cancer	Family cancer history
*ERCC5* (NM_000123)	c.3004C > T	p.Q1002X	Pathogenic	*TCF7L2* (NM_001146274) c.407A > G p.H136R VUS	BRAF p.V600E	70	c-PTC	I	NED	No	Yes
*TCF7L2* (NM_001146274)	c.450 + 1G > T	p.?	Pathogenic	*AMER1* (NM_152424) c.187T > G p.D6E VUS	No	37	c-PTC	L	NED	No	No
*APC* (NM_000038)	c.5365G > T	p.V1789L	Likely Pathogenic	*PMS2* (NM_000535) c.1004A > G p.N335S Conflicting interpretation of pathogenicity	BRAF p.V600E + TERT (C228T)	65	c-PTC	I	NED	No	Yes^b^
*BMPR1A* (NM_004329)	c.1416G > T	p.E472D	Likely Pathogenic	*FEN1* (NM_004111) c.802T > G p.Y268D VUS + *ERCC5* (NM_000123) c.56C > T p.P19L VUS	BRAF p.V600E + TERT (C228T)	65	c-PTC	I	SED	No	No
*TGFBR2* (NM_001024847)	c.1710C > G	p.D570E	Likely Pathogenic	No	NRAS p.Q61R	27	mixed PTC	L	NA	NA	NA
*MLH1* (NM_000249)	c.1013A > G	p.N338S	Conflicting interpretation of pathogenicity	No	BRAF p.V600E	45	c-PTC	L	NED	No	No
*MLH3* (NM_014381)	c.1343C > T	p.P448L	Conflicting interpretation of pathogenicity	No	BRAF p.V600E	38	c-PTC	L	NED	No	Yes
*APC* (NM_000038)	c.8182G > A	p.V2728M	VUS	*MYC* (NM_002467.6) c.1085C > T p.S362F VUS	KRAS p.G12V	25	c-PTC	L	NA	NA	NA
*ARID1A* (NM_006015)	c.1385A > G	p.Y462C	VUS	*POLE* (NM_006231) c.2171C > T p.A724V VUS	BRAF p.V600E	64	c-PTC	I	NED	No	Yes
*BMPR1A* (NM_004329)	c.56C > T	p.S19F	VUS	No	No	34	fv-PTC	L	NA	NA	NA
*OGG1* (NM_016828)	c.971T > C	p.F324S	VUS	No	NRAS p.Q61R	53	fv-PTC	L	NED	No	No
*PCNA* (NM_002592)	c.304 oG > A	p.V102I	VUS	No	NRAS p.Q61K	52	fv-PTC	L	NA	NA	NA
*PMS2* (NM_000535)	c.113C > T	p.A38V	VUS	*ERCC5* (NM_000123) c.844G > A p.V282I VUS	BRAF p.V600E	47	OXYPHILIC-PTC	I	NED	No	Yes
*POLE* (NM_006231)	c.1735C > T	p.R579C	VUS	No	BRAF p.V600E	48	c-PTC	L	BED	No	Yes
	c.6673C > T	p.R2225C	VUS	No	BRAF p.V600E	49	c-PTC	I	NA	NA	NA
*XPC* (NM_004628)	c.1616A > G	p.E539G	VUS	No	BRAF p.V600E	37	c-PTC	I	NED	No	No
	c.2173C > T	p.R725W	VUS	No	BRAF p.V600E	47	c-PTC	I	NA	NA	NA
	c.2404G > A	p.G802S	VUS	No	No	61	fv-PTC	L	NED	No	Yes

*BED* biochemical evidence of disease, *I* intermediate, *L* low, *NA* not applicable, *NED* not evidence of disease, *SED* structural evidence of disease, *VUS* variants of uncertain significance

^a^The clinical interpretation was performed according to Varsome, wIntervar, LOVD, and Clinvar databases

^b^Including thyroid cancer

Following ACMG classification, the identified germline SNVs included 2 pathogenic (8%), 3 likely pathogenic (12%), 17 variants of unknown significances (68%), and 3 (12%) variants having conflicting interpretation of pathogenicity, being simultaneously annotated as likely benign, VUS and likely pathogenic. The two pathogenic variants were detected in two patients both burdened by a classic PTC, with a tumor size falling into the upper 95% CI of the median. *ERCC Excision Repair 5* (*ERCC5*, NM_000123) c.3004C > T is a missense variant introducing a stop codon (p. Q1002X). The second pathogenic variants is located in the splice region of the *Transcription factor 7-like 2* (*TCF7L2*) exon 4 (NM_001146274: c.450 + 1G > T). Three likely pathogenic variants have been identified in four different patients. *APC* (NM_000038) c.5365G > T is a missense variant causing a valine to leucine change in position 1789 of APC protein sequence (p.V1789L). Alteration in the bone morphogenetic protein receptor type 1A (*BMPR1A*, NM_004329) c.1416G > T is a missense variant which causes a glutamic acid to be replaced by an aspartic acid in the amino acid chain (p.E472D) in the protein kinase domain. *Transforming growth factor beta receptor 2* (*TGFBR2*, NM_001024847) c.1710C > G is a missense variant which causes an aspartic acid to be replaced by a glutamic acid in the amino acid chain (p.D570E). Three SNVs annotated with conflicting interpretation of pathogenicity were detected in *MutL Homolog 1* (*MLH1*), *MutL Homolog 3* (*MLH3*), and *PMS1 homolog 2, mismatch repair system component (PMS2)*, all involved in the DNA mismatch repair (MMR) pathway: *MLH1* (NM_000249) c.1013A > G (p.N388S); *MLH3* (NM_014381) c.1343C > T (p. P448L); *PMS2* (c.1004A > G; p.N335S).

We did not find any statistically significant association between the presence of a germline variant and any clinical characteristics, treatment outcome or somatic mutational status of the analyzed patients, except for more frequent lymph-node metastases observed in absence of germline alterations (*p* = 0.0236) (Table 3).

**Table 3:** Association between the presence of a germline variant and patients’ clinical characteristics

	Germline variants		*p* value
	No	Yes	
Patients (no.)	24	18	
Sex^a^			
Female	14 (67%)	9 (50%)	0.2915^e^
Male	7 (33%)	9 (50%)	
Average age at diagnosis (m ± SD)^a^	42 ± 18	47.5 ± 13.5	
Minimum	22	25	0.9722^f^
Maximum	84	70	
Histology			
PTC-CT	19 (79%)	12 (67%)	0.7738^e^
PTC-FV	3 (12%)	4 (22%)	
PTC-MIXED	1 (4.5%)	1 (5.5%)	
Other	1 (4.5%)	1 (5.5%)	
Tumor size (mm), (m ± SD)^b^	12 ± 14.3	14.5 ± 8.7	
Minimum	5	3	0.4935^f^
Maximum	50	38	
T stage			
T1	6 (25%)	9 (50%)	0.35^e^
T2	2 (8%)	1 (6%)	
T3	15 (63%)	8 (44%)	
T4	1 (4%)	0 (0%)	
Presence of metastasis			
Lymph node (N)	13 (54%)	3 (17%)	0.0236^g^
Distant (M)	0 (0%)	1 (5%)	0.439^g^
ATA risk of recurrence at diagnosis			
Low	7 (29%)	10 (56%)	0.1819^e^
Intermediate	16 (67%)	8 (44%)	
High	1 (4%)	0 (0%)	
Outcome^c^			
NED	10(72%)	10(84%)	0.6534^e^
BED	3 (21%)	1 (8%)	
SED	1 (7%)	1 (8%)	
Somatic mutation (Total)^d^	15 (62%)	15 (83%)	0.18^g^
* BRAF*	13 (54%)	9 (50%)	0.137^e^
* BRAF* + *TERT*	0 (0%)	2 (11%)	
* RAS*	1 (4%)	4 (22%)	
* TERT*	1 (4%)	0 (0%)	

*BED* biochemical evidence of disease, *NED* not evidence of disease, *SED* structural evidence of disease

^a^Three missing values

^b^One missing value

^c^16 without follow-up

^d^TERT was tested on 17 cancer tissues

^e^Chi-square test

^f^Mann–Whitney *U* test

^g^Fisher’s exact test

## Discussion

The molecular basis of PTC development and progression is not completely understood. Despite the strong evidence of heritability, only a small subset of alterations has been convincingly implicated in PTC pathogenesis so far [14]. The best characterized markers of PTC aggressiveness are the somatic *BRAF* p.V600E mutation, which occurs in ~40% of all PTCs [15] and the coexisting BRAF p.V600E and *TERT* promoter mutations associated with PTC-specific mortality [16].

In this study, we used a targeted NGS panel to investigate the molecular profiles of a cohort of PTC specimens, identifying point mutations in 78.5% of samples. We once again confirmed the high prevalence of the BRAF p.V600E somatic mutation in classic PTC and of KRAS p.G12V, NRAS p.Q61R, and p.Q61K somatic mutations in the follicular variants of PTC. The prevalence of *TERT* promoter somatic mutations in PTC from this study was consistent with that reported by TCGA (9%) [16].

We did not find any somatic mutations in the other genes associated with cancer aggressiveness analyzed. Unpredictably, 45% of the identified mutations resulted to be germline. Following ACMG classification, 20% of pathogenic/likely pathogenic and 68% of variant of unknown significances were identified.

We highlighted a nonsense mutation in *ERCC5* gene in a 70-years-old female patient who also harbors the BRAF p.V600E somatic mutation (VAF = 30%). *ERCC5*, also known as *XPG*, encodes a structure-specific endonuclease that has multiple functions during nucleotide excision repair, a DNA repair pathway triggered by ultraviolet (UV)-induced damage [7]. The amino acid change falls into the C-terminal PCNA-binding domain, whose function is crucial for repair efficiency [14]. Alterations affecting its coding sequence or its mRNA levels can impair DNA repair resulting in genomic instability and carcinogenesis. Homozygous or compound heterozygous mutation in *ERCC5* are associated to the xeroderma pigmentosum complementation group G (XPG), a rare disease characterized by photosensitive erythema, keratoses and skin and eye disorders due to a high sensitivity to UV radiations. Indeed, *ERCC5* has been associated with an increased risk of developing skin tumors (basalomas, squamous cell carcinomas, melanomas). Interestingly, a patient’s relative was affected by a uterine carcinoma, that is also reported to be also be associated to the ERCC5 p.Q1002X. Indeed, previous studies have linked *ERCC5* SNVs to soft tissues cancer susceptibility, including endometrial and thyroid [7, 17]. The tumor was positive for the BRAF p.V600E mutation.

In a 37-years-old female patient, a splice-site mutation has been identified in *TCF7L2*. TCF7L2 is an important component of the Wnt signaling pathway. *TCF7L2* (previously reported as *TCF-4*) is a transcription factor that interacts with β-catenin in the nucleus inducing the expression of target genes, including CCND1 and c-MYC, involved in cellular proliferation, evasion of apoptosis, and tissue invasion and metastasis [18]. Loss of function mutations in *TCF7L2* have been associated to both prostate [18] and colon cancer [19] susceptibility and to an increased risk of Type II diabetes [20]. We identified a variant in the splice region of *TCF7L2*, which alters RNA splicing reducing the amount of the functional codified protein. Accordingly, the genome aggregation database (gnomAD) identifies *TCF7L2* as a strong loss-of-function intolerant gene. The pLI (probability of being loss-of-function intolerant), representing the intolerance toward protein-truncating variation, is 1, strengthening the concept of being extremely intolerant toward loss-of-function alterations. Thus, in silico data corroborate the possibility that this alteration to be strongly deleterious.

Mutations in *APC* are known to be causative of familial adenomatous polyposis, colon hepatocellular, and gastric carcinoma together with desmoid tumors. We found an APC p.V1789L substitution in a 65-years-old female patient in whose medical history, in addition to the PTC, a previous duodenal ulcer and breast fibroadenoma were also reported. Moreover, her family history turned to be positive for TC, liver, and genitourinary cancers. This variant was not previously reported and is not enlisted in any population databases (i.e., GnomAD). In contrast, this amino acid appears to be only partially phylogenetically conserved, hindering the possibility to clearly annotate it as a deleterious variant. It should be noted that the patient turned to be also positive for the BRAF p.V600E and the TERT c.‐124C > T (C228T).

Alteration in *BMPR1A* have been strongly associated to an increased susceptibility to the juvenile polyposis syndrome and to colorectal cancer [21]. We identified a p.E472D substitution in BMPR1A in a 45-years-old male patient. Although this amino acid change does not suggest the abolition of the protein kinase domain functionality, this variant was not found in the gnomAD database, suggesting a possible impact in protein activity. Literature regarding the role of *BMPR1A* in thyroid carcinogenesis are still scanty. It should be noted that the tumor was positive for BRAF p.V600E (VAF = 15%) and *TERT* C228T mutations and was reclassified in the ATA high risk category at the last follow-up.

Interestingly the two PTCs harboring both the *TERT* and *BRAF* mutations have a very different prognosis (Table 2) suggesting that the different germline mutational status may play a role in the modulation of cancer aggressiveness.

A missense mutation was identified in a 27-years-old male patient who also carries the NRAS p.Q61R mutation (VAF = 46.5%). Alteration in *TGFBR2* have been associated to the Lynch syndrome, squamous cell carcinoma of the esophagus and the connectivopathies Loeys-Dietz and type-2 Marfan syndromes. TGFBR2 p.D570E falls in a hot-spot region where most missense variants are annotated as pathogenic. Notwithstanding the aminoacidic change occurs between two negative-charged residues, the position is quite phylogenetically conserved.

Moreover, three SNVs were classified having conflicting interpretation of pathogenicity, being simultaneously annotated as likely benign, VUS and likely pathogenic. Indeed, inactivating mutations in both *MLH1* and *PMS2* are associated to the Lynch syndrome, a heritable condition associated with a greatly increased risk of colorectal, endometrial, stomach, and ovarian cancers together with tumors of the small intestine, the biliary tract, brain, ureters, and renal pelvis [22]. MLH1 p.N388S is located in a hot-spot region where 24 variants, all pathogenic, have been identified. Nevertheless, this SNV has been previously assessed, suggesting a potential lack of impact in the protein function [23, 24]. MLH3 p.P448L is simultaneously annotated as benign and VUS and is not present in gnomAD database. Besides that, this residue is not strongly conserved and the Varsome database annotated this variant as a polymorphism. Since this SNP was never been published before nor in vitro experiments have been performed to assess its impact on MLH3 functions, we are unable to strongly associate this alteration to papillary thyroid carcinogenesis. Finally, the PMS2 p.N335S was detected in the same PTC sample that also harbored the APC p.V1789L. This residue is highly conserved during phylogenesis and in silico predictors strongly suggest its pathogenic effect. Therefore, the co-presence of the two alterations, the complexity of the familial history and the fact that *PMS2* has a much lower penetrance for Lynch Syndrome than the other MMR genes [25], prevents us from assessing whether it is deleterious or not. More data, including the segregation analysis in affected families, should be collected before definitely annotate these alterations.

The only statistically significant association we found with the patient’s clinical features is between the presence of a germline variation and a less frequent lymph-node involvement. However, patients with a germline mutation tended to be male and to co-occur with a somatic mutation. Unfortunately, the patient’s cohort analyzed is admittedly too small to reach reliable conclusions.

The main finding of our study is the frequent involvement of alterations in DNA repair genes in PTC predisposition. This is in accordance with recent findings that reported the presence of germline mutations in DNA repair genes in a concurrent composite mucoepidermoid carcinoma and papillary thyroid carcinoma suggesting their putative role in the predisposition to TC development [26]. Interestingly, all the pathogenic and putatively pathogenic mutations found in our PTC cohort are not present in the major population databases (i.e., gnomAD). In our opinion this result is extremely relevant. Since typical PTC-related pedigrees do not often follow Mendelian inheritance but, instead, are small with irregular transmission of the cancer phenotype, the most widely used approach for the investigation on PTC genetics relied on genome-wide association studies. Despite the efforts, this kind of studies has failed to explain the “missing” heritability of PTC [7]. Indeed, our results tell a different story: the predisposition to PTC may be due to very rare germinal variants, not investigated in polygenic and gene-environment interactions-based studies.

Regardless, the results presented here should be observed only as proof of concept and must therefore be validated through replication in larger populations and implemented with familial segregation analysis. Moreover, our data are derived from a targeted panel while the use of massive technologies such as the whole exome sequencing will surely help to shed better light on familial predisposition to PTC.

In conclusion, notwithstanding the limitations in our approach, our data support the hypothesis that rare germline variants in genes involved in DNA repair are accountable for PTC susceptibility, rather than genome-wide-based risk scoring.

## Supplementary information

Supplementary Information
