# Supplementary material for: Rare germline variants in DNA repair-related genes are accountable for papillary thyroid cancer susceptibility
Source: Endocrine. 2021 Apr 5;73(3):648–57. doi: 10.1007/s12020-021-02705-1 (PMC8325654; doi:10.1007/s12020-021-02705-1)
Supplement: Supplementary file 1 — Supplementary Information [file 12020_2021_2705_MOESM1_ESM.docx]

## Supplementary Information

### Supplementary Table 1. List of genes included in the targeted NGS panel and related pathways

| Gene ID | **RefSeq ID** | **Tumor progression** | **DNA repair** |
| --- | --- | --- | --- |
| AMER1 | NM_152424 | Wnt pathway |  |
| APC | NM_000038 | Wnt pathway |  |
| APEX1 | NM_001244249 |  | Base excision repair |
| ARID1A | NM_006015 |  | Chromatin remodeling |
| BAX | NM_004324 | Apoptosis pathways |  |
| BMPR1A | NM_004329 | TGF-beta pathway |  |
| BRAF | NM_004333 | RAS/MAPK pathway |  |
| CDKN2A | NM_058195 | Cell cycle regulation |  |
| CTNNB1 | NM_001904 | Wnt pathway |  |
| ERBB2 | NM_004448 | PI3K-Akt pathway |  |
| ERCC1 | NM_001983 |  | Nucleotide excision repair |
| ERCC4 | NM_005236 |  | Nucleotide excision repair |
| ERCC5 | NM_000123 |  | Nucleotide excision repair |
| FBXW7 | NM_033632 | Notch Pathway |  |
| FEN1 | NM_004111 |  | Base excision repair |
| GNAS | NM_080425 | RAS pathway |  |
| IGF2 | NM_001127598 | PI3K-Akt pathway |  |
| KRAS | NM_033360 | RAS/MAPK pathway | MGMT-mediated DNA repair pathway |
| MGMT | NM_002412 |  |  |
| MLH1 | NM_000249 |  | Mismatch repair pathway |
| MLH3 | NM_001040108 |  | Mismatch repair pathway |
| MDG | NM_001015052 |  | Base excision repair |
| MSH2 | NM_000251 |  | Mismatch repair pathway |
| MSH6 | NM_000179 |  | Mismatch repair pathway |
| MUTYH | NM_001128425 |  | Base excision repair |
| MYC | NM_002467 | PI3K-Akt pathway |  |
| NRAS | NM_002524 | RAS/MAPK pathway |  |
| OGG1 | NM_016820 |  | Base excision repair |
| PARP1 | NM_001618 | Apoptosis pathways | Nucleotide excision repair |
| PCNA | NM_182649 |  | Nucleotide excision repair |
| PIK3CA | NM_006218 | PI3K-Akt pathway |  |
| PMS2 | NM_000535 |  | Mismatch repair pathway |
| POLB | NM_002690 |  | Base excision repair |
| POLD1 | NM_001256849 |  | Nucleotide excision repair |
| POLE | NM_006231 |  | Nucleotide excision repair |
| PTEN | NM_000314 | PI3K-Akt pathway |  |
| RET | NM_020975 | RAS/MAPK pathway |  |
| RUNX3 | NM_004350 | Wnt pathway |  |
| SOX9 | NM_000346 | Wnt pathway |  |
| STK11 | NM_000455 | RAS/MAPK pathway |  |
| TCF7L2 | NM_001146274 | Wnt pathway |  |
| XPA | NM_000380 |  | Nucleotide excision repair |
| XPC | NM_004628 |  | Nucleotide excision repair |
| XRCC1 | NM_006297 |  | Base excision repair |
| TGFBR2 | NM_001024847 | NF-kB pathway |  |
| TIMP3 | NM_000362 | RAS/MAPK pathway |  |
| TP53 | NM_001276760 | Apoptosis pathways |  |
